# Ethnicity-specific microbiome in early childhood caries: a functional perspective of oral biofilm

**DOI:** 10.1128/msystems.01787-25

**Published:** 2026-04-23

**Authors:** Kuei-Ling C. Hsu, Tara N. Furstenau, Isaac Shaffer, Mark D. Macek, Robert K. Ernst, Viacheslav Y. Fofanov

**Affiliations:** 1 Department of Orthodontics and Pediatric Dentistry, School of Dentistry, University of Maryland 89015, Baltimore, Maryland, USA; 2 School of Informatics, Computing, and Cyber Systems, Northern Arizona University 731587, Flagstaff, Arizona, USA; 3 Department of Dental Public Health, School of Dentistry, University of Maryland 89015, Baltimore, Maryland, USA; 4 Department of Microbial Pathogenesis, School of Dentistry, University of Maryland 89015, Baltimore, Maryland, USA; The University of Hong Kong, Hong Kong, Hong Kong

**Keywords:** early childhood caries, oral microbiome

## Abstract

National surveillance data show persistent racial and ethnic disparities in early childhood caries (ECC), but the underlying causes of these differences have not been determined. This study examined both functional and taxonomic differences in ECC-related microbial activity between two high-risk groups of children: African American (AA) and Latin American Hispanic (LAH). Metatranscriptomic profiling of paired non-caries and caries plaque revealed consistent population-level shifts in gene expression and enabled species-level attribution of metabolically active microbes in caries lesions. A core set of well-established cariogenic organisms was consistently present and highly over-expressed in caries of both groups, including *Streptococcus mutans*, *Veillonella parvula*, *Propionibacterium acidifaciens*, and *Lactobacillus rhamnosus*. Beyond identifying the core organisms and functions active in lesions, we have also made two significant observations. First, the active communities in the two groups have substantially diverged: 4,900+ genes across 413 Kyoto Encyclopedia of Genes and Genomes Orthology (KO) groups were consistently (25%+ of samples) over-expressed in AA children, and 6,500+ genes across 382 KOs were consistently (57% of samples) over-expressed in LAH children. This reproducibility across multiple samples indicates robust group-level differences rather than random variation or single-sample effects. Second, although AA and LAH children exhibited similar functional responses to caries (sharing 1,392 KOs), these shifts were expressed by different bacterial species, indicating that distinct taxa may occupy similar metabolic niches in different groups. Taken together, these findings suggest that there is no single universal caries-associated microbiome; instead, a shared cariogenic core is necessary, but differences among the non-core taxa and their functional activity may be key to understanding ECC disparities.

**IMPORTANCE:**

The disparity in tooth decay among young children has long been demonstrated in national surveillance data. While various factors including family, culture, access to health insurance, and medical infrastructure have been studied, the global transcriptomic perspective remains underexplored. Employing RNA-Seq technology, we examine functional and taxonomic differences in caries-associated microbial activity between two high-risk populations. Besides a core set of well-established cariogenic organisms, we observed significant and consistent differences in the active microbial communities between these two high-risk populations, African American (AA) and Latin American Hispanic (LAH) children. In AA children, *Pseudopropionibacterium propionicum* and *Cardiobacterium hominis* consistently showed the highest caries-related gene expression. In contrast, among LAH children, *Propionibacterium acidifaciens*, *Selenomonas* sp., *Rothia dentocariosa*, *Atopobium parvulum*, and *Streptococcus sanguinis* were the primary drivers of gene expression in caries lesions. By identifying the unique microbial mechanisms and pathways active in each population, we can better define the core factors required for caries development and uncover how differences in microbial function contribute to persistent disparities.

## INTRODUCTION

Early childhood caries (ECC) is a severe and aggressive form of dental caries in children younger than 6 years of age. In the United States, it is the most common chronic disease among children and is more prevalent in socioeconomically disadvantaged populations (1). ECC has far-reaching consequences, including increased risk of additional caries lesions in both primary and permanent dentitions (2, 3), reduced appetite due to pain with eating, delayed physical growth and development, lack of sleep, irritability, low self-esteem, as well as poor attendance and performance in school (4). Children with ECC visit emergency rooms more often and have higher hospitalization rates (5–7), which increases the cost of medical care. If left untreated, fatal orofacial infections or brain abscesses can occur (8, 9). Moreover, young children suffering from severe ECC often require surgical intervention for full mouth rehabilitation, which can cost up to $25,000 per case (10, 11). In 2009, the total dental expenses for US children aged 5–17 years were approximately $20 billion and accounted for 17.7% of all healthcare expenses, with approximately 40% of dental costs paid out of pocket, compared to 17% for medical care (12).

Caries prevalence and the composition of caries-associated biofilm vary by race and ethnicity (13–18). Although socioeconomic and behavioral determinants influence disparities in ECC, distinct differences in microbiota composition across racial and ethnic groups are evident. Hispanic children have the highest prevalence of dental caries (57.1%), followed by Non-Hispanic Black children (48.1%); however, Non-Hispanic Black children have the highest prevalence of untreated dental caries (17.1%), with Hispanic children following at 13.5% (1, 19). Studies have shown ethnicity-specific clustering of microbial communities in saliva and subgingival biofilms, which enables the development of predictive microbial signatures for ethnicity (14, 16). Significant differences in alpha and beta diversity have also been reported, with specific taxa from supragingival samples uniquely associated with ethnic groups among children affected by caries (20).

The presence of acidogenic biofilm bacteria is a key factor in caries development. *Streptococcus mutans* has long been recognized as a primary cariogenic species in humans and has been shown to induce dental caries in gnotobiotic animal models (21–23). *S. mutans* is critical in forming a stable acidic biofilm that recruits and maintains a cariogenic microbial community (24). The virulence of *S. mutans* is correlated with its capacity to synthesize extracellular glucan polymers from dietary sucrose that promote colonization and biofilm accumulation on tooth surfaces. Additionally, *S. mutans* can metabolize diverse carbohydrates into acids that demineralize enamel and enhance conditions for acid-tolerant bacteria to persist and proliferate within harsh acidic environments (25, 26). However, epidemiological evidence suggests that although *S. mutans* is strongly linked with and often necessary for the development of ECC (27, 28), it is often detected in caries-free children; therefore, its presence alone is not sufficient to predict disease (29–33).

In addition to *S. mutans*, strong evidence now shows that many other bacteria also play roles in caries patients and are therefore potentially linked to oral diseases and include *Lactobacillus*, *Bifidobacterium*, *Scardovia*, *Parascardovia*, *Actinomyces*, *Fusobacterium*, *Porphyromonas*, *Selenomonas*, *Bacteroides*, *Haemophilus*, *Veillonella*, *Leptotrichia*, and *Thiomonas* (28, 34). These findings support the ecological hypothesis (35) introduced in the modern era, which suggests that imbalances of the oral bacteria/microbes can lead to the initiation and progression of oral disease (36). Although little is understood about the ecological and functional roles of these bacteria, it is increasingly clear that the cariogenic activity of an oral biofilm is most likely a complex and polymicrobial etiology rather than caused by a single species (37–40). Therefore, it is important to investigate the microbial community to understand how intricate relationships and synergies impact the oral microbiome and cause disease.

This observational, cohort study investigated transcriptionally active microbial communities in ECC lesions to determine how their functional roles differ in two high-risk groups: African American (AA) and Latin American Hispanic (LAH) children. Taxonomic variations across racial and ethnic populations have been reported in prior studies; however, it remains unclear how these differences influence microbial gene expression and metabolic activity. The observation that similar disease outcomes occur despite differences in microbial composition across racial and ethnic groups suggests that distinct bacterial taxa may occupy equivalent functional niches, driving caries through alternative but convergent metabolic pathways. By investigating both shared and ethnicity-specific microbial functions, we identified key functional processes that are required for caries progression, providing a clearer understanding of factors influencing ECC disparities.

## MATERIALS AND METHODS

### Study population and sample collection

For the study, children younger than 6 years of age at the pediatric dental clinic at the University of Maryland School of Dentistry were enrolled. After a thorough explanation of the risks, benefits, and alternatives to the parent/legal guardian and confirmation of the eligibility, consent was obtained from the parent/legal guardian. The children were medically healthy, had not taken antibiotics or used corticosteroids in the preceding 3 months, and had no acute dental infection, such as swelling or abscess, at the time of the sample collection (2018–2020). Dental plaque from teeth with and without caries was collected using sterile toothpicks. Caries lesions were characterized by obvious visual cavitation at the enamel and into the dentin. Radiographs were not used for additional identification. A single, board-certified examiner performed the clinical examination. Non-cavitated lesions (decalcification or white spot lesions) were not sampled. The plaque samples were immediately transferred to RNAlater (0.5 mL per sample) and mixed before frozen at −80°C until further analyses were performed.

In addition to plaque sample collection, descriptive data were also collected, including the patient’s age, sex, and dental condition, including dentition, the total number of decayed, missing, and filled primary teeth (dmft), and surfaces (dmfs). Race and ethnicity information was self-reported by the parents and used the categories defined by National Institutes of Health. Children identified as Hispanic were of Latin American origin and are therefore described as Latin American Hispanic (LAH). Dietary information was collected for each participant, including the daily frequency of snack and beverage consumption, the most frequently consumed snack and drink items, and whether the overall diet was primarily carbohydrate based. Snack and beverage frequency was categorized as equal to or greater than or less than three times per day, reflecting the predefined response options used during data collection. Snacks were classified as either sugary/sticky or other, and beverages were categorized as sugary/acidic or other.

### RNA extraction and next-generation sequencing

Dental plaque samples were lysed using bead beating (FastPrep instrument with 0.1 mm silica spheres) in the presence of acid phenol. Following chloroform extraction, total RNA was precipitated using isopropanol and resuspended in DEPC-treated water. After DNase treatment to remove residual DNA, RNA was purified using the RNeasy kit (Qiagen). To prepare sequencing libraries (Azenta), rRNA was depleted using QIAGEN FastSelect 5S/16S/23S kit (Qiagen, Hilden, Germany), and RNA was processed using the NEBNext Ultra II RNA Library Preparation Kit for Illumina following standard guidelines (NEB, Ipswich, MA, USA). Libraries were multiplexed and run on an Illumina HiSeq-4000 to yield 2 × 150 bp paired-end data.

### Quality controls and computational subtraction of human reads

Sequence quality was assessed using the fastp software package (41, 42). Average quality scores and adapter contamination levels were within acceptable thresholds, with quality scores of 30 or higher and adapter contamination of 5% or lower. The only notable exception was the caries lesion sample from patient 75, which exhibited ~10% adapter contamination. Contaminating human reads were identified by aligning reads to the human genome (version GRCh38) using the Bowtie2 software package (43) with default parameters and computationally subtracting matched reads using samtools package (44) with default parameters.

### Taxonomic composition analysis

To identify the species composition of the samples, we performed taxonomic classification of the sequence reads using the previously published MTSv software package (45). MTSv is an alignment-based metagenomic classifier that is highly accurate and produces fewer false positives than other rapid taxonomic classifiers. The reference sequences were downloaded from the NCBI’s GenBank Database (accessed on 28 October 2019). All reference sequences without a valid (species level or below) taxonomic classification or those labeled as “environmental” samples were discarded; all subspecies taxonomic classifications (e.g., strain level) were relabeled as their parent species classification. The final reference database included sequences for 4,530 bacteria and 255 archaea. Reads were aligned against the reference database with MTSv using a seed size of 14 bp with a 2 bp interval between seeds and a minimum requirement of 4 seed hits for initiating a full alignment.

### Gene reference database

A custom gene database was constructed using reference gene sequences from the most prevalent species identified across all samples. This was defined as the union of the top 150 species with at least 1,000 assigned reads (Table S4). All gene reference sequences associated with the top 284 species were downloaded from the Kyoto Encyclopedia of Genes and Genomes (KEGG) database (https://www.genome.jp/kegg/, accessed on August 2020). For 50 species lacking gene data in KEGG, annotations from the closest species within the same genus were substituted. This yielded 892,674 gene sequences representing 7,940 KEGG orthologs, which were used to build a species-level gene classification database.

### Differential expression analysis for comparative metatranscriptomics

The metatranscriptomic reads were assigned using MTSv with a custom gene reference database that allowed us to classify reads by gene and species (45). To improve sensitivity, we only considered alignments in regions unique to each gene for each species. This allowed us to reduce false assignments in cases where sequence similarity between genes from different species is high. DESeq2 software for R (46) was used to estimate fold changes. The “approximate posterior estimation for generalized linear models” (apeglm) (47) model and software for estimating the more stable log2 fold change shrinkage from Zhu et al. were used (47). The full linear model used for the matched caries lesion and non-caries plaque samples was ~ Subject + Race + Caries status + Race:Caries status, which captured the sample pairings and the effects of population.

We defined significantly differentially expressed (SDE) genes as those with an adjusted *P*-value ≤ 0.05 (Benjamini and Hochberg method [48]) and a shrunken log2 fold change greater than 0.58 (log_2_ 1.5). For each racial group, SDE genes were extracted using a contrast comparing caries lesion samples to paired non-caries plaque samples within that group. Genes meeting significance criteria in both groups were labeled “Shared,” while those significant in only one group were labeled “AA only” or “LAH only.” Genes that were SDE in one group but in the other group had a similar shrunken log_2_ fold change (within 0.5), but fell below the cutoff for significance, were labeled as “Shared” to avoid counting differences that were simply an artifact of cutoff placement. Functional categories were assigned to genes based on the KEGG functional hierarchy for the KEGG Orthology (KO) associated with each gene. For KOs with multiple functional classifications, we chose the one that appeared most commonly in the data set and the more specific one in the case of a tie.

## RESULTS

### Study overview and population demographics

**Fig 1:**
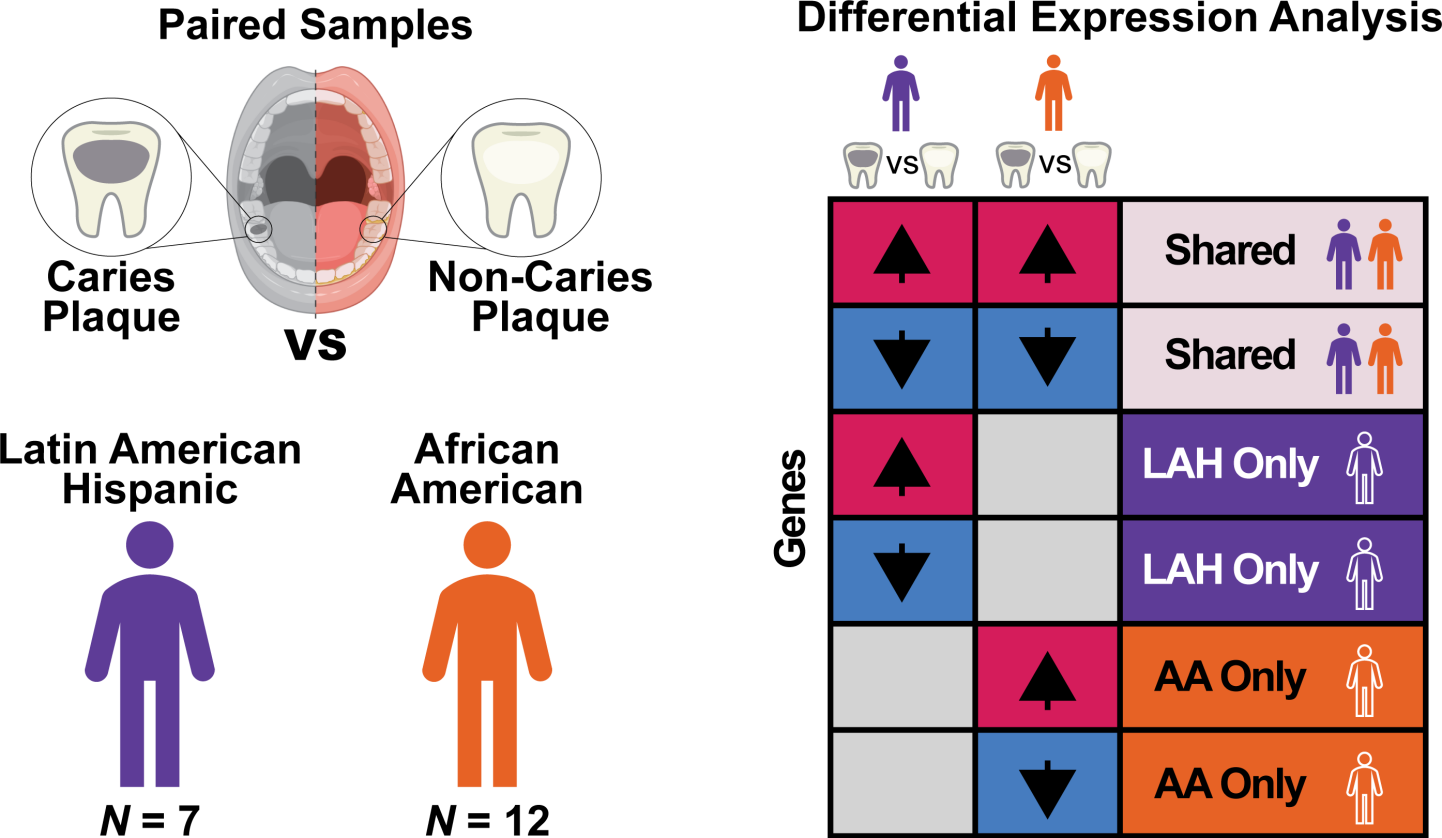
Overview of study design and differential expression analysis. Paired dental plaque samples were collected from caries lesions and non-caries sites in children with ECC from two racial/ethnic groups: AA (*n* = 12) and LAH (*n* = 7). Metatranscriptomic sequencing was performed on all samples, and differential expression analysis was conducted to compare caries vs. non-caries plaque within individuals. Statistical contrasts were used to identify significantly differentially expressed genes within each racial/ethnic group, enabling detection of both shared and group-specific microbial and functional responses associated with caries.

The overview of the study design and differential expression analysis of genes is illustrated in Fig. 1. In this study, paired dental plaque samples were collected from caries and non-caries lesions from individual AA (*n* = 12) and LAH (*n* = 7) subjects with ECC (Table S1—ECC cohort). This cohort (*n* = 19) was 58% female and 42% male, with a mean age of 4.4 years. The caries status of the subjects was determined by dmft and dmfs that were decayed, missing (due to decay), and filled. The mean dmft and dmfs among all subjects were 8.2 and 18.9, respectively. The mean dmft and dmfs values were 6.8 and 15.3 among AA children and 10.7 and 25.1 among LAH children, respectively. LAH children had a significantly higher dmft (*P* = 0.04), while differences in dmfs were not statistically significant (using two-sample unpaired *t*-tests). No significant differences were observed between groups in reported dietary habits (Fisher’s exact test), including snacking frequency (*P* = 0.17), drink frequency (*P* = 0.60), consumption of primarily sugary and/or sticky snacks (*P* = 0.17), primarily sugary and/or acidic drinks (*P* = 0.11), or a carbohydrate-dominant diet (*P* = 0.62). However, given the group sizes, the study was powered to detect only large effects, and the non-significant dietary comparisons should be interpreted cautiously.

### Sequencing depth and quality

The total RNA extracted from plaque samples ranged from 100 ng to 1,000 ng, and high sequencing coverage was achieved with low adapter contamination (Table S2). Species accumulation curves for the ECC and caries-free cohorts (Fig. S1), as well as the AA and LAH (Fig. S2), indicated that the number of bacterial organisms reached a point of diminishing returns (at approximately 32 samples in the ECC cohort and 22 samples in the caries-free cohort), suggesting that the sequencing depth and the number of samples we collected sufficiently captured most dominant taxa.

### Bacterial species abundance

The bacterial species composition of each sample was determined by alignment-based taxonomic classification of sequence reads using the MTSv software package (45). Table S3 shows the species with the largest fold change (differences in gene expression between the paired plaque samples within the ECC cohort). In 13 of 19 children, the median fold change for *Streptococcus mutans* reads was nearly 500-fold higher in caries plaque. Other classical cariogenic bacterial species, including *Lactobacillus rhamnosus* and *Streptococcus parasanguinis*, were also identified in higher abundance in caries plaque. Interestingly, there were non-classical cariogenic bacterial species, including *Parascardovia denticolens*, *Olsenella* sp*.* oral taxon 807, *Olsenella uli*, *Selenomonas sputigena*, and *Prevotella denticola*, consistently more abundant in the caries plaque of at least seven ECC subjects.

### Consistent microbial activity associated with caries across AA and LAH children

To investigate species-level transcriptional changes associated with caries, we compared gene expression between paired caries and non-caries plaque samples from the same children. Using model contrasts, we identified genes that were SDE in caries for both the AA and LAH children and evaluated the extent to which these gene expression patterns were shared or specific to each racial/ethnic group. Overall, SDE genes from each group exhibited consistent expression patterns across individuals, with an average of 36% of AA samples and 63% of LAH samples showing at least twofold change in expression consistent with each SDE gene.

**Fig 2:**
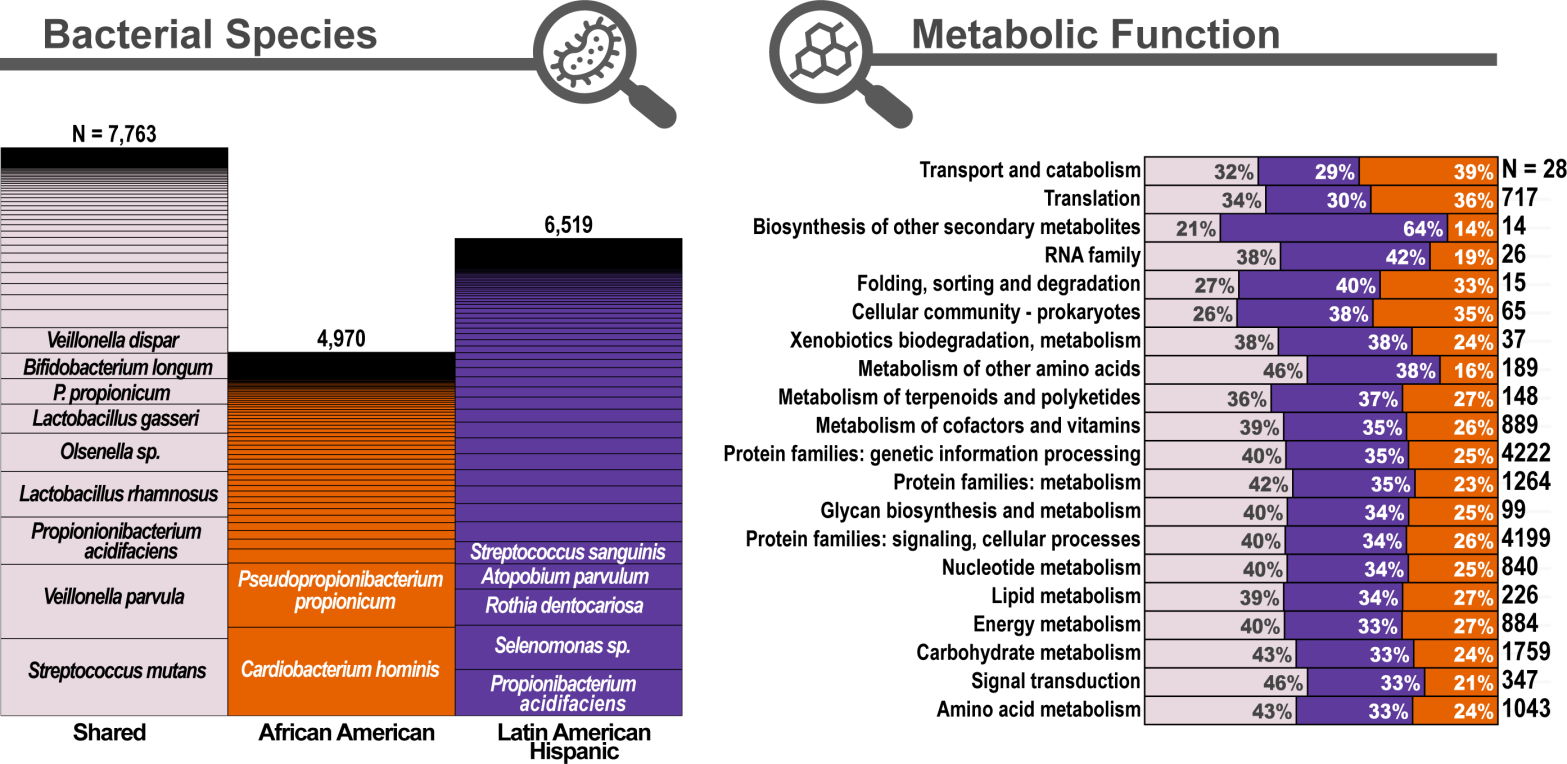
African American and Latin American Hispanic children display unique taxonomic and functional microbial pathways contributing to caries development. In this study, we compared gene expression between paired caries-active and non-caries plaque within the same children using a novel analysis tool that provides species-level gene resolution. By analyzing genes that were significantly differentially expressed in caries plaque in African American and Hispanic children, we found similar patterns of gene expression from organisms typically associated with caries formation, but we also found many differences between the two groups. We conclude that while several species act as required core species in both groups, they each follow a functionally and taxonomically unique path to caries progression.

**Fig 3:**
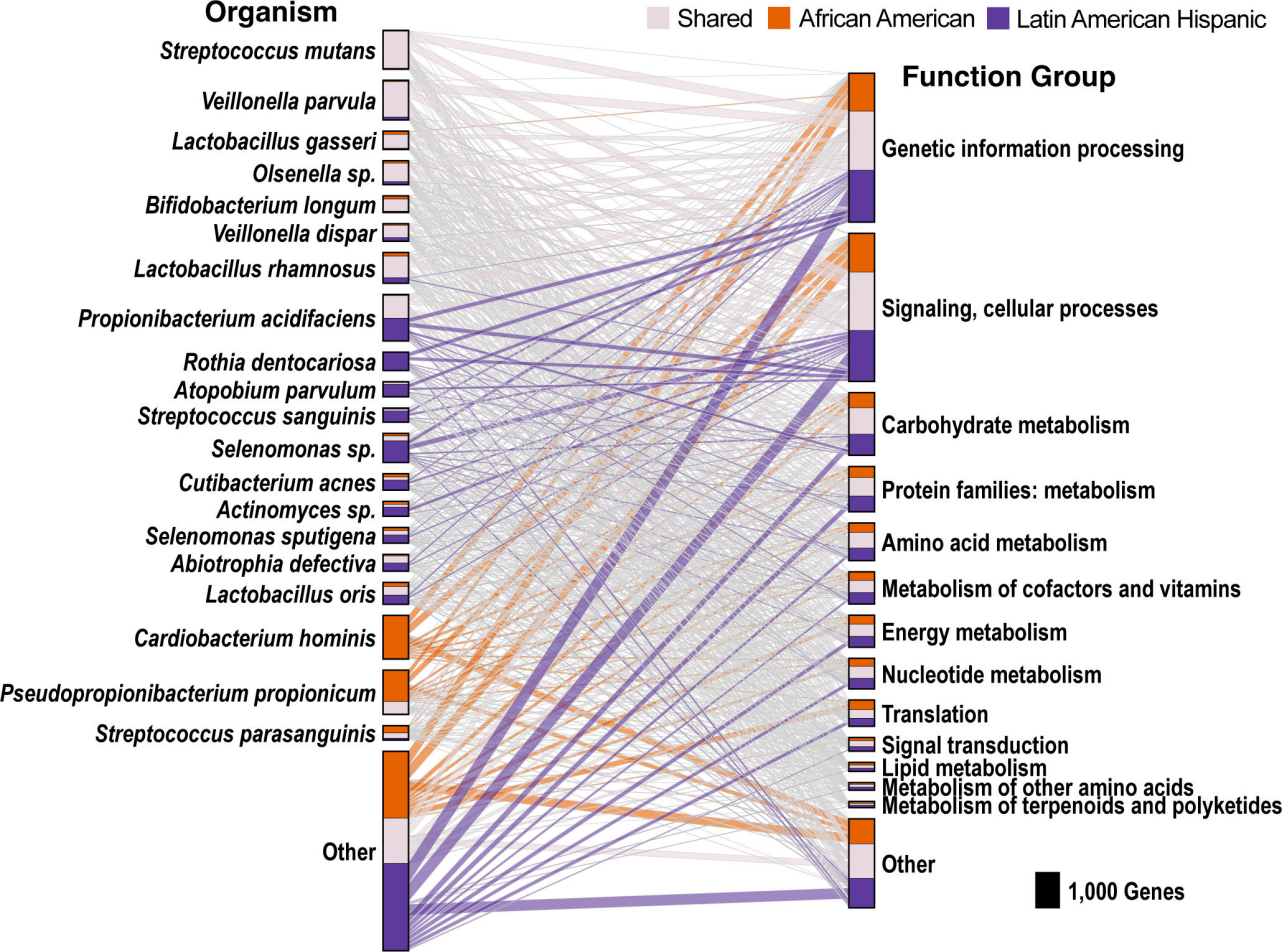
Key cariogenic species showed consistent and significant differential gene expression in caries lesions versus non-caries plaque in both AA and LAH groups. The figure shows the significantly differentially expressed genes in caries vs. non-caries plaque, highlighting the top organisms and functional groups. Gray lines indicate genes that were differentially expressed in both the AA and LAH groups. Purple and orange lines indicate genes that were only significantly differentially expressed in either the AA or the LAH group, respectively.

**Fig 4:**
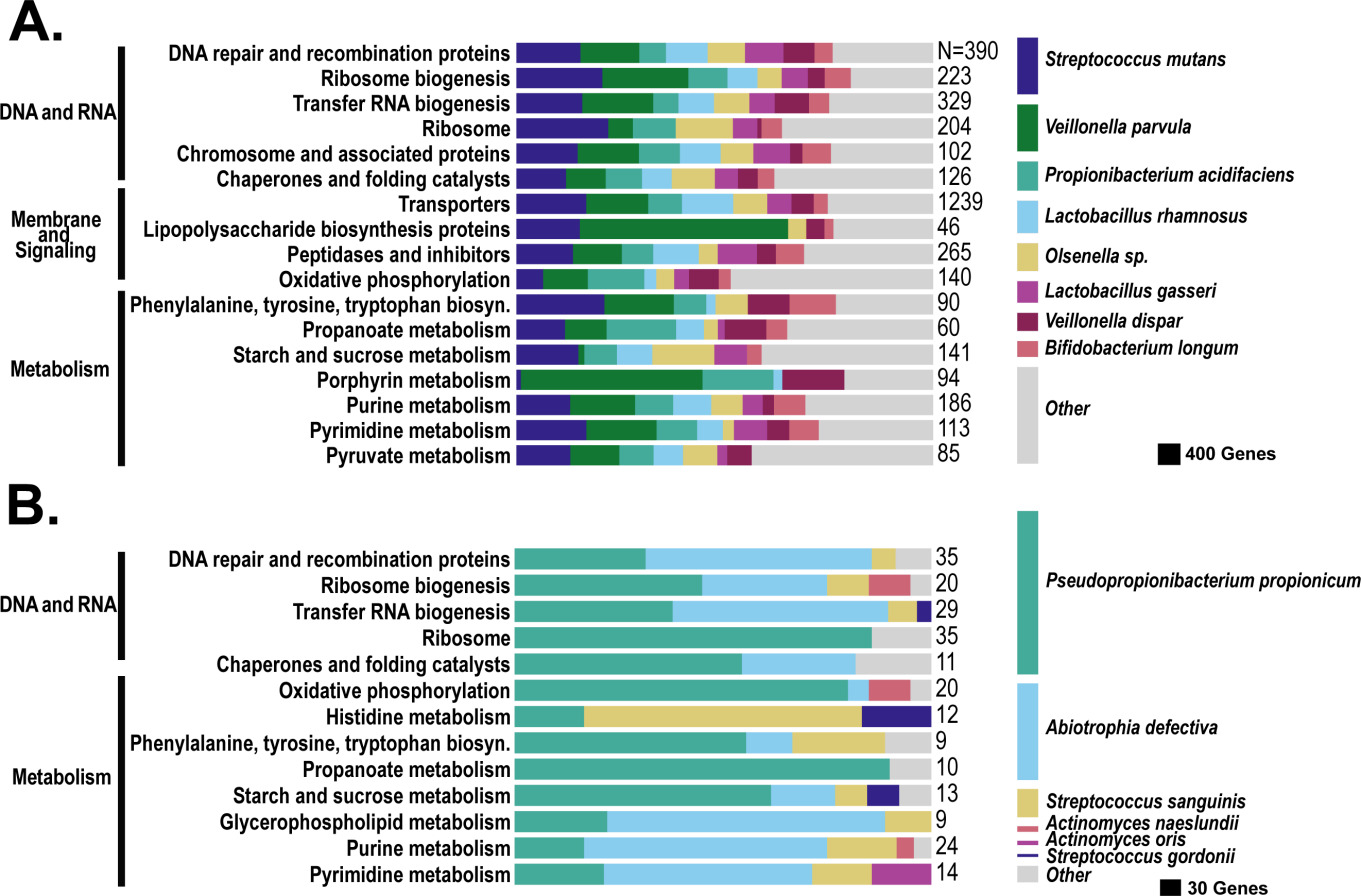
Gene expression in caries lesions shows upregulation of *S. mutans* and cariogenic species and downregulation of acid-neutralizing and caries-inhibiting functions in both AA and LAH groups. The figure shows the distribution of species associated with genes that were significantly upregulated (**A**) or downregulated (**B**) in caries plaque in both the African American and Hispanic groups. The values on the right side of each chart are the total number of genes in each bar. The legend on the right is proportional to the overall number of genes associated with the upregulated (*N* = 6,967) or downregulated (*N* = 676) genes. The functional categories shown are those that had the highest percent of shared KEGG orthology.

The AA and LAH children shared 7,763 genes from 2,186 KOs that were SDE in caries compared to non-caries plaque (Fig. 2). Of these genes, 6,967 were upregulated, 676 were downregulated, and the remaining 120 were regulated in opposite directions in AA and LAH children. Shared transcriptional changes were primarily driven by well-established ECC-associated bacteria, including *Streptococcus mutans*, which made up 15% of shared upregulated genes, *Veillonella parvula* (15%), *Propionibacterium acidifaciens* (9%), lactobacilli (*Lactobacillus rhamnosus*, 9%; *Lactobacillus gasseri*, 6%; *Lactobacillus oris*, 4%), *Olsenella* sp. (8%), and *Bifidobacterium longum* (5%; Fig. 2 to 4A). In contrast, shared downregulated genes were predominantly attributed to health-associated species (49, 50), including *Pseudopropionibacterium propionicum* (51%), *Abiotrophia defectiva* (30%), and *Streptococcus sanguinis* (9%; Fig. 4B).

Among the top shared upregulated pathways (Fig. 4A), starch and sucrose metabolism, lipopolysaccharide biosynthesis, and porphyrin metabolism stood out as functionally relevant to caries pathogenesis, supporting increased acid production and biofilm formation. These functional shifts were predominantly contributed by *S. mutans*, *Veillonella*, and *Lactobacillus*. Conversely, downregulated pathways in caries included oxidative phosphorylation, amino acid metabolism, and alkali generation (Fig. 4B). The suppression of these functions in caries suggests a potential shift away from biofilm growth (51).

### Differences in active organisms and functions between African American and Latin American Hispanic children with ECC

**Fig 5:**
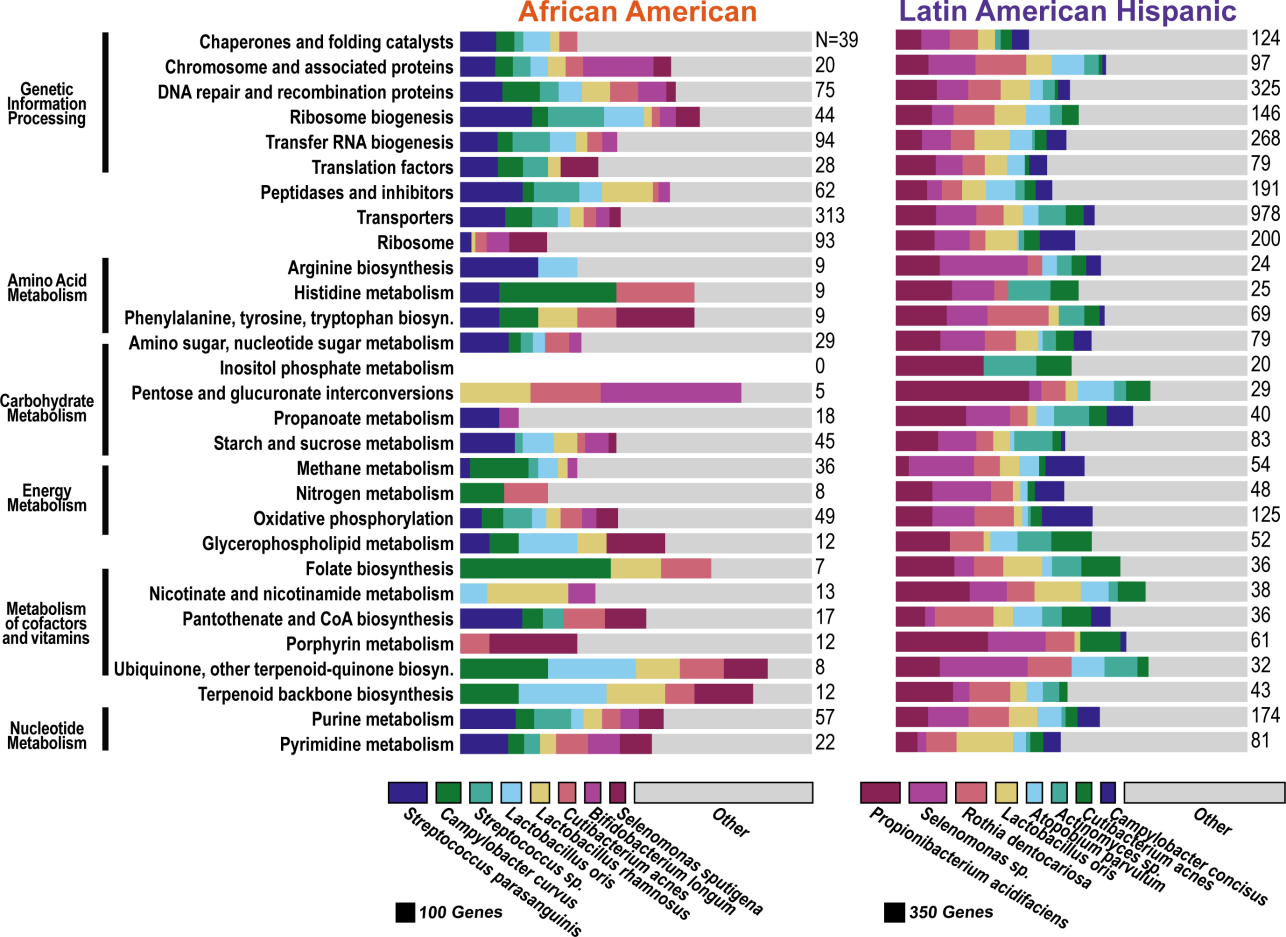
Functional and taxonomic differences in significantly upregulated genes between caries and non-caries plaque that were unique to the AA or LAH groups. Bar charts show the distribution of genes significantly upregulated in either the AA group (left, total *N* = 1,746, not all shown in chart) or the LAH group (right, total *N* = 5,683, not all shown in chart). Rows represent top functional categories, and colors indicate the relative contributions of the top eight species per group. Numbers on the right of each bar indicate the total number of SDE genes represented. Bar widths in the legend at the bottom are scaled to reflect the total number of upregulated genes from each species across all functional categories. The top species associated with the upregulated genes differ between the groups, except for *Cutibacterium acnes*, which has upregulated genes in each group, but they were not the same genes.

**Fig 6:**
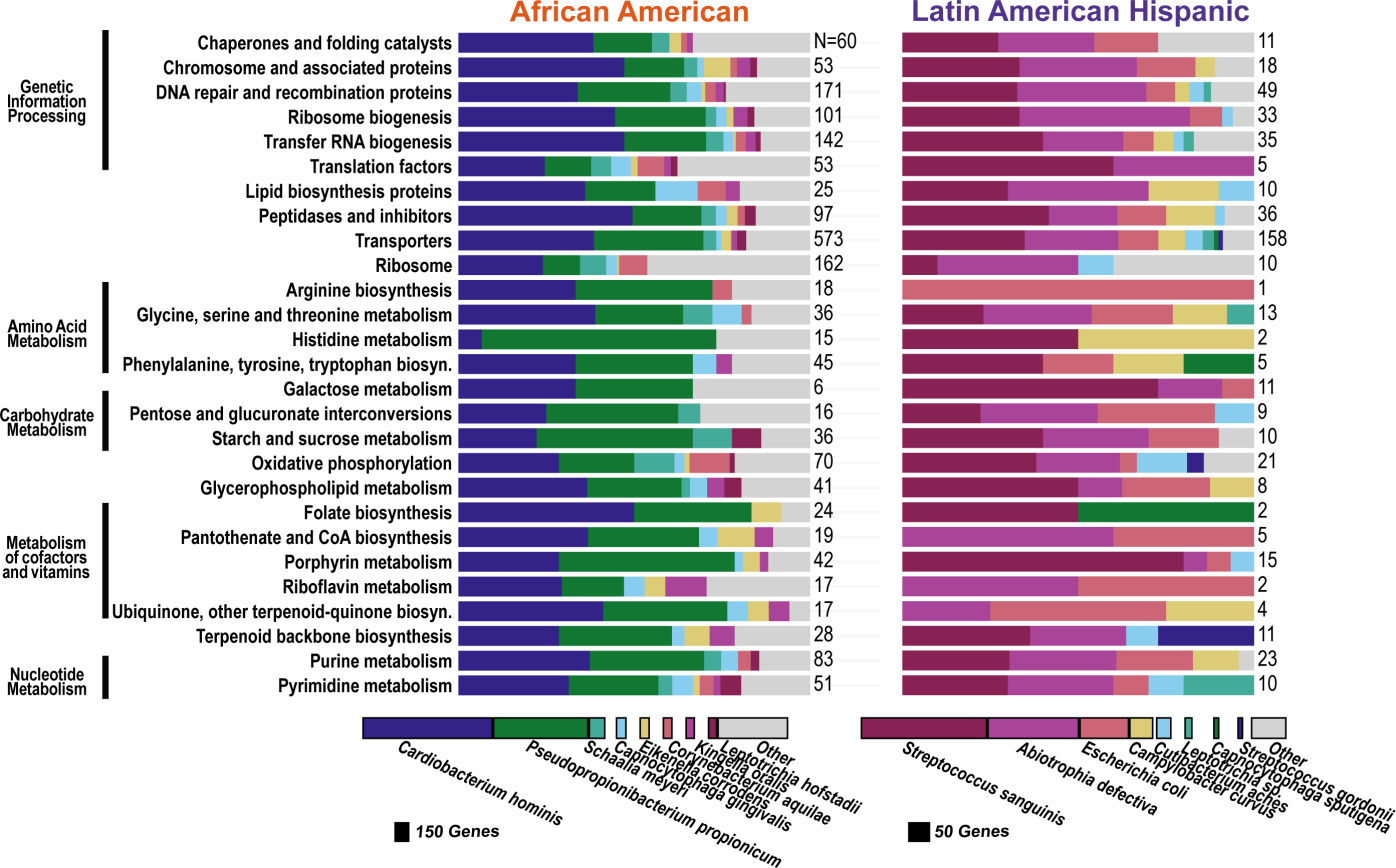
Functional and taxonomic differences in significantly downregulated genes between caries and non-caries plaque that were unique to the AA or LAH groups. Bar charts show the distribution of genes significantly downregulated in either the AA group (left, total *N* = 3,224, not all shown in chart) or the LAH group (right, total *N* = 836, not all shown in chart). Rows represent top functional categories, and colors indicate the relative contributions of the top eight species per group. Numbers on the right of each bar indicate the number of SDE genes represented. Bar widths in the legend at the bottom are scaled to reflect the total number of downregulated genes from each species across all functional categories.

Although many of the SDE genes were shared between the AA and LAH groups, there were consistent group-specific differences in the organisms and gene functions expressed in caries compared to non-caries plaque. We identified 4,970 genes (from 2,106 KOs) that were uniquely SDE in AA ECC children and 6,519 genes (from 2,236 KOs) uniquely SDE in LAH ECC children (Fig. 2). Genes from *Streptococcus parasanguinis* were the most prominently upregulated in caries among the AA children, along with *Campylobacter curvus*, *Lactobacillus*, *B. longum*, and *Selenomonas sputigena* (Fig. 5). These species have been linked to caries and include several acid-utilizing and/or producing species (30, 52–58). In AA children, most (65%) of the unique SDE genes were downregulated and largely associated with *Pseudopropionibacterium propionicum* and *Cardiobacterium hominis* (Fig. 6), suggesting reduced abundance or activity of these species in caries-affected plaque. Notably, both species have been linked to healthy, caries-free oral states in previous studies (49, 50).

The genes uniquely differentially expressed in LAH children were predominantly upregulated (87%) and primarily associated with *Propionibacterium acidifaciens*, *Selenomonas* sp*.*, *Rothia dentocariosa*, *Atopobium parvulum*, and *Streptococcus sanguinis* (Fig. 5)—some of which have cariogenic potential (49, 53, 55, 56). The predominance of upregulated cariogenic species in LAH children, compared with the predominance of downregulated genes from health-associated species in AA, suggests that caries in AA children may be more closely linked to the loss of protective species, whereas caries in LAH children may be driven more by the introduction or increased activity of cariogenic taxa. Uniquely downregulated genes in LAH were less common and were primarily associated with *S. sanguinis*, *A. defectiva*, and *Escherichia coli*. Although *E. coli* is not typically considered part of the oral microbiota, highly specific read alignments to *E. coli* were detected in all samples, albeit at low relative abundance. Greater than twofold changes in expression of *E. coli* SDE genes were observed in six of the six LAH children, indicating that this pattern was not driven by a single individual. Given the young age of the children in the study population, its presence may reflect colonization via the fecal-oral route or oral-gut microbial exchange. The observed differential expression in caries plaque may indicate that *E. coli* is responding to the more acidic environment.

**Fig 7:**
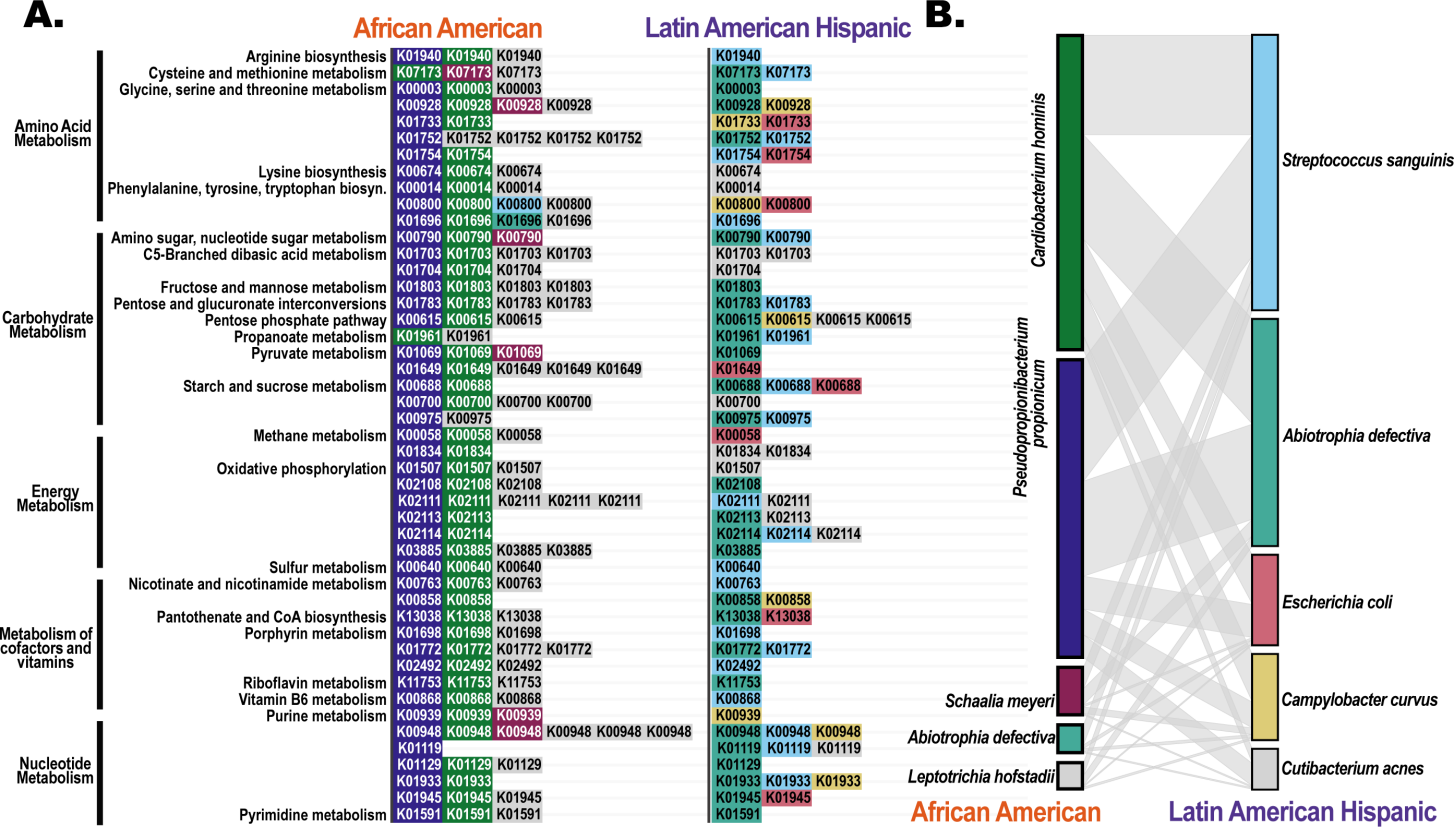
Evidence of functional convergence by distinct microbial taxa in AA and LAH groups. (**A**) KOs that were SDE in both groups but were associated with entirely distinct species, suggesting similar functional activity across communities. Each bar represents a KO, and colors indicate the species contributing to that KO in each group. (**B**) Linkage diagram showing the species most frequently associated with similar patterns of differential expression across groups. Lines show the number of times the species shared KOs across the two groups. Species color coding is consistent between panels A and B.

To determine whether the group-specific gene expression patterns reflected functional convergence with distinct organisms filling similar roles, we next examined whether the same KOs were differentially expressed across groups despite being contributed by different taxa. Among KOs that were upregulated in both groups, 889 out of 2,128 (41.8%) differed in species attribution, including 99 (4.7%) with no species overlap between groups. For KOs downregulated in both groups, the divergence was even stronger: 503 of 596 (84.4%) differed in species attribution, and 465 (78%) exhibited no overlap between groups. Figure 7 shows a subset of the functional categories of downregulated genes where there was no overlap between the groups and indicates which species were performing similar functional behaviors in each group. Conversely, there were 795 differentially expressed KOs that were unique to one group: 413 (52%) in AA and 382 (48%) in LAH. These group-specific KOs suggest the presence of functional activity that was not mirrored by other organisms in the other group. Together, these results indicate that while some bacterial functions appear conserved or are fulfilled by different taxa in each population, other functions may be uniquely active in caries in one group and absent in the other, pointing to both shared and divergent ecological processes in the caries state.

## DISCUSSION

The ecological plaque hypothesis (35) has shifted our understanding of how microbial communities drive oral disease, emphasizing that an imbalance in the total oral microbiome, in contrast to the presence of specific pathogens, contributes to an environment that favors disease. This ecological shift promotes acid metabolism, lowers environmental pH, and promotes enamel demineralization. By directly sequencing RNAs from plaque biofilm in both caries and non-caries teeth in high-risk minority children, our study captured the metabolic shifts that occur between the healthy and disease state, providing new insights into both shared and ethnicity-specific microbial mechanisms driving ECC.

### Strengths and limitations of our experimental design and metatranscriptomic analysis approach

Using paired samples of plaque from caries and non-caries sites in the same individual minimized inter-individual variation and accounted for demographic and sample-type influences, providing a powerful framework to identify microbial changes linked to caries, yielding higher species-level resolution than protein-centric methods (59, 60). This approach enabled direct linking of differential gene expression to specific taxa rather than averaging across genera. We utilized our previously developed transcript assignment tool, which performs sensitive full nucleotide alignments, resulting in more accurate species-level assignments with fewer false positives compared to similar tools (45).

Our study included 38 samples from 19 participants, which were sufficient for performing the differential analysis (61), all from lower-income households in the Baltimore metropolitan area, providing valuable insights within this focused community. While controlling for geographic and socioeconomic factors strengthened the internal validity, our findings may not be generalizable to broader populations. We sequenced more than 50 million reads per sample with a high percent remaining after human subtraction, and the species accumulation curves indicated that a saturation point was reached, so we are confident that our results captured the diversity of transcripts present in these samples.

### A core set of cariogenic organisms is active in caries plaque in both African American and Latin American Hispanic children

Our findings reconfirm that the well-established cariogenic bacteria, including *S. mutans* and *Lactobacillus gasseri*, are highly expressed in caries lesions and are shared across both the AA and LAH groups. In addition to the classical cariogenic bacteria, we also identified other bacterial species, such as *Veillonella parvula*, *Olsenella* sp., and *Bifidobacterium longum*, which other studies have identified as potentially cariogenic (27, 37–39). Many upregulated porphyrin metabolism genes and lipopolysaccharide biosynthesis genes were associated with *Veillonella parvula*, suggesting a potential influence on bacterial membrane structure and host-relevant biochemical pathways. In addition, most of the shared downregulated genes were contributed by three species: *Pseudopropionibacterium propionicum* (51%), *Abiotrophia defectiva* (30%), and *Streptococcus sanguinis* (9%). These organisms were previously reported to be more abundant in caries-free children (49, 62, 63). Most of the shared downregulated genes were associated with *Pseudopropionibacterium propionicum* and were related to oxidative phosphorylation and propanoate metabolism. This suggests that energy production through oxidative phosphorylation and utilization of propanoate may be less efficient in caries (Fig. 3B). The expression of genes associated with alkali-generating pathways in *S. sanguinis* was downregulated in caries samples, which suggests a reduced ability to buffer the pH of the biofilm (51). Other studies (64–66) have reported the interaction between fungi (*Candida albicans*) and *S. mutans* in promoting the development of ECC. However, in our study, *Candida albicans* was not consistently detected within the cavity or matched plaque samples—only 2 of 19 cavity lesion samples (AA and LAH child) and 1 of 19 matched plaque samples had more than 1 million reads unambiguously assigned to *C. albicans*.

### Compositional and functional differences in the cavity microbiome between African American and Latin American Hispanic children

While both populations shared a core cariogenic microbiome, we observed marked and consistent differences in the active microbial species between the AA and LAH groups. In AA children, caries-associated gene expression was dominated by downregulation in *Pseudopropionibacterium propionicum* and *Cardiobacterium hominis*. In LAH children, caries-associated gene expression was dominated by a more taxonomically diverse set of species, including highly upregulated genes in *Propionibacterium acidifaciens*, *Selenomonas* sp*.*, *Rothia dentocariosa*, *Lactobacillus oris*, *Actinomyces* sp*.*, *Atopobium parvulum*, and *Campylobacter concisus*, many of which have been associated with increased caries risk (49, 55, 56). Species largely associated with downregulated genes in LAH children included *S. sanguinis*, *A. defectiva*, and *E. coli*, which have been linked to healthy, caries-free microbiomes or may be antagonistic toward *S. mutans* (57, 58). A recent study by Cho et al. (53) reported that *Selenomonas sputigena* could act as a pathobiont mediating spatial structure and potentially contribute to biofilm virulence in ECC children. These findings are consistent with our data, with *S. sputigena* being upregulated in caries in the AA ECC group and other *Selenomonas* species upregulated in the LAH ECC group.

### Distinct microbial communities fill equivalent functional niches in African American and Latin American Hispanic children

Despite differences in microbial composition, we observed functional redundancy in caries-associated microbiomes between AA and LAH children. Notably, 21% of differentially expressed genes were shared across populations but were expressed by different bacterial species, suggesting that distinct microbial communities can perform analogous roles in disease progression. The fact that different bacterial species drive similar gene expression patterns suggests that functional niches within the caries biofilm remain consistent across populations, even when the taxonomic composition varies. This highlights the importance of studying microbial function rather than relying solely on taxonomic composition, as distinct microbial communities can sustain cariogenic activity through parallel metabolic pathways. While these parallels suggest conserved mechanisms of disease progression, further research is needed to identify where functional pathways diverge, revealing novel, population-specific mechanisms that may contribute to ECC disparities.

### Potential role of diet in shaping differences in microbial function between African American and Latin American Hispanic children

Although no striking differences in the dietary patterns between AA and LAH children, diet remains a plausible contributor to the microbiome differences identified. Additionally, the limited statistical power and coarse resolution of dietary assessments may have concealed moderate differences in dietary exposures that influence microbial community structure and function. Regular intake of sugary or acidic beverages and snacks between meals is strongly linked to increased caries risk and disease progression (67–71). National data show racial and ethnic differences in sugar-sweetened beverage intake, with African American youth generally consuming a higher share of daily calories than Hispanic peers, though both groups exceed recommended limits (72). Moreover, acculturation and access likely shape diet and oral health: dietary patterns in Hispanic families shift with acculturation (73, 74). In this study, approximately half of both the AA and LAH groups reported consuming sugary or acidic drinks and snacks three or more times daily, and their diets were predominantly carbohydrate based. The absence of detectable dietary differences between the groups reflects the brief nature of the questionnaire, which captured only broad patterns rather than detailed intake, and limited power due to the small sample size at the subject level.

### Factors impacting the interpretation of results

Beyond diet, differences between groups may also reflect variation in the clinical characteristics of caries lesions. Previous studies have shown that the depth and progression of caries lesions influence microbial composition due to nutrient availability at different layers of the caries biofilm. *Streptococcus mutans*, *Bifidobacterium*, and *Streptococcus parasanguinis* have been found in significantly higher abundance in cavitated enamel and dentin lesions (54), which aligns with our findings. In addition, studies have reported higher levels of *Veillonella dispar* and *Scardovia wiggsiae* in severe early childhood caries (S-ECC) cases compared to both less severe ECC and caries-free children (63, 75, 76). In our study, all participants except one were categorized as S-ECC, and we observed high expression of *V. dispar*, consistent with prior findings. Notably, LAH children had a significantly higher number of affected teeth (dmft) compared to AA children; however, the surfaces affected (dmfs) were not significantly different. While lesion-level variation was not directly assessed in our study, the consistent classification of S-ECC across participants suggests broad similarity in disease severity. However, the observed differences in dmft raise the possibility of underlying variation in lesion progression, duration, or other unmeasured clinical or behavioral factors. These differences may contribute to the microbial variation detected between groups and warrant further investigation.

### Conclusion

Our findings provide evidence that ECC is driven by both shared and population-specific microbial mechanisms, reinforcing the need to move beyond a single-species model of caries development. While a core group of cariogenic bacteria, including *S. mutans*, was consistently present and active across both populations, distinct microbial communities were found among AA and LAH groups. There were equivalent functional roles performed by different species present in both the AA and LAH groups, which suggests that different species may fill common functional niches in caries. Our findings provide conserved and divergent functional pathways that contribute to ECC in two different racial/ethnic groups. Further research should aim to expand these findings in larger, more diverse cohorts and incorporate other perspectives, such as oral hygiene, access to fluoridated water/supplements, and dental utilization, to further refine our understanding of the microbial mechanisms responsible for ECC disparities.

## Data Availability

The RNA sequencing reads are available through NCBI’s Sequence Read Archive under BioProject no. PRJNA1024640. Analysis scripts and Tables S4 and S5 are available through FigShare at 10.6084/m9.figshare.25439143. The STORMS checklist is available through FigShare at https://figshare.com/ndownloader/files/60428786.

## SUPPLEMENTAL MATERIAL

Supplemental material msystems.01787-25-s0001.pdf Figures S1 and S2 and Tables S1-S3.

Table S4 msystems.01787-25-s0002.xlsx Genes that were significantly differentially expressed in African American and Hispanic groups.

Table S5 msystems.01787-25-s0003.xlsx Taxa included in the gene reference database downloaded from KEGG.

ASM does not own the copyrights to Supplemental Material that may be linked to, or accessed through, an article. The authors have granted ASM a non-exclusive, world-wide license to publish the Supplemental Material files. Please contact the corresponding author directly for reuse.
