# Supplementary material for: Ethnicity-specific microbiome in early childhood caries: a functional perspective of oral biofilm
Source: mSystems. 2026 Apr 23;11(5):e01787-25. doi: 10.1128/msystems.01787-25 (PMC13185588; doi:10.1128/msystems.01787-25)
Supplement: Supplemental material — Figures S1 and S2 and Tables S1-S3. [file msystems.01787-25-s0001.pdf]

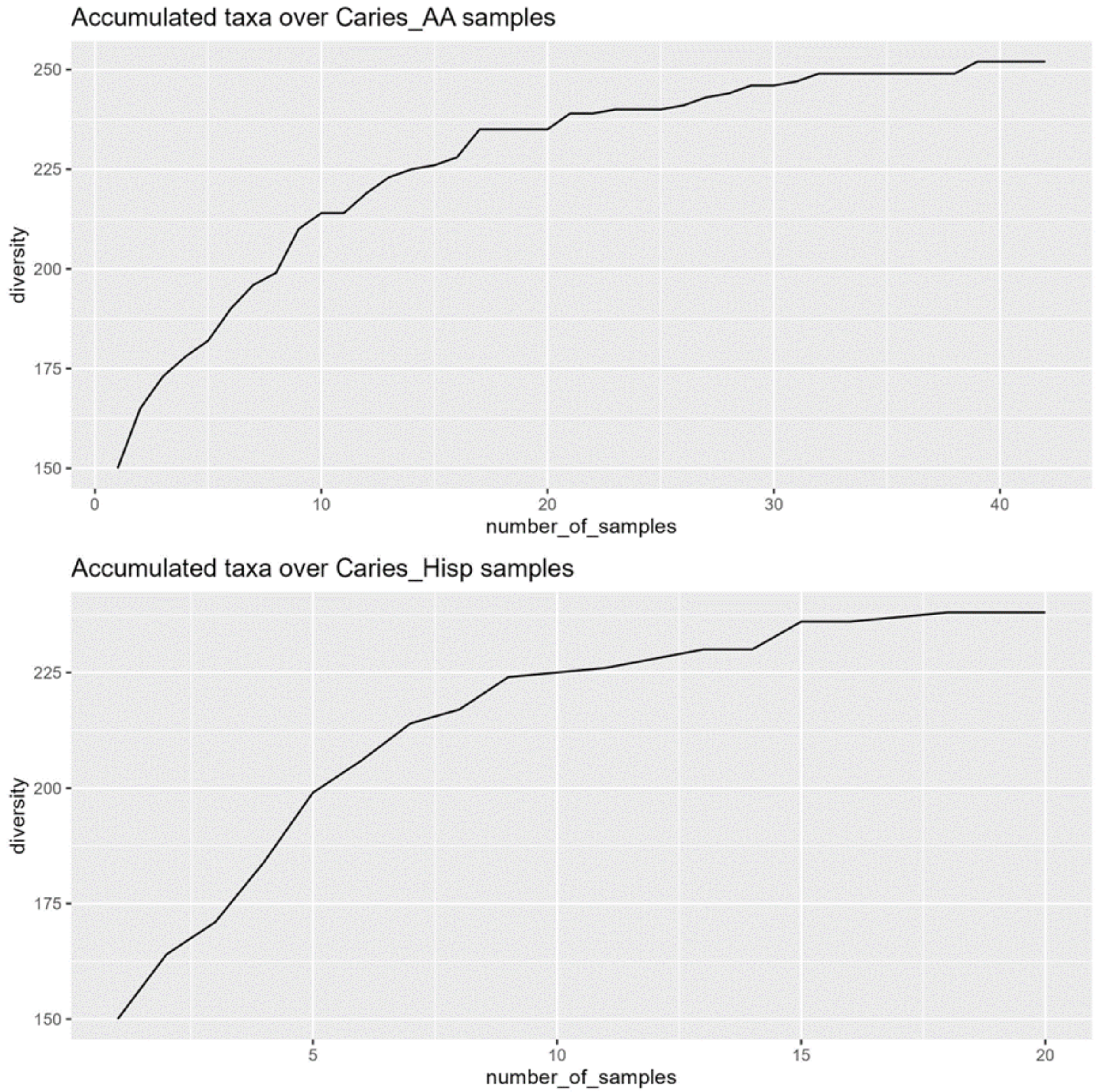

Figure S1. Species accumulation curve from taxonomic composition analysis of Latin American Hispanic vs African American samples. The cumulative number of new taxa (at the species level) identified with each new sample from the African American (top) and Latin American Hispanic (bottom) samples. A species was considered present if there were at least 1,000 reads that uniquely aligned to the species.

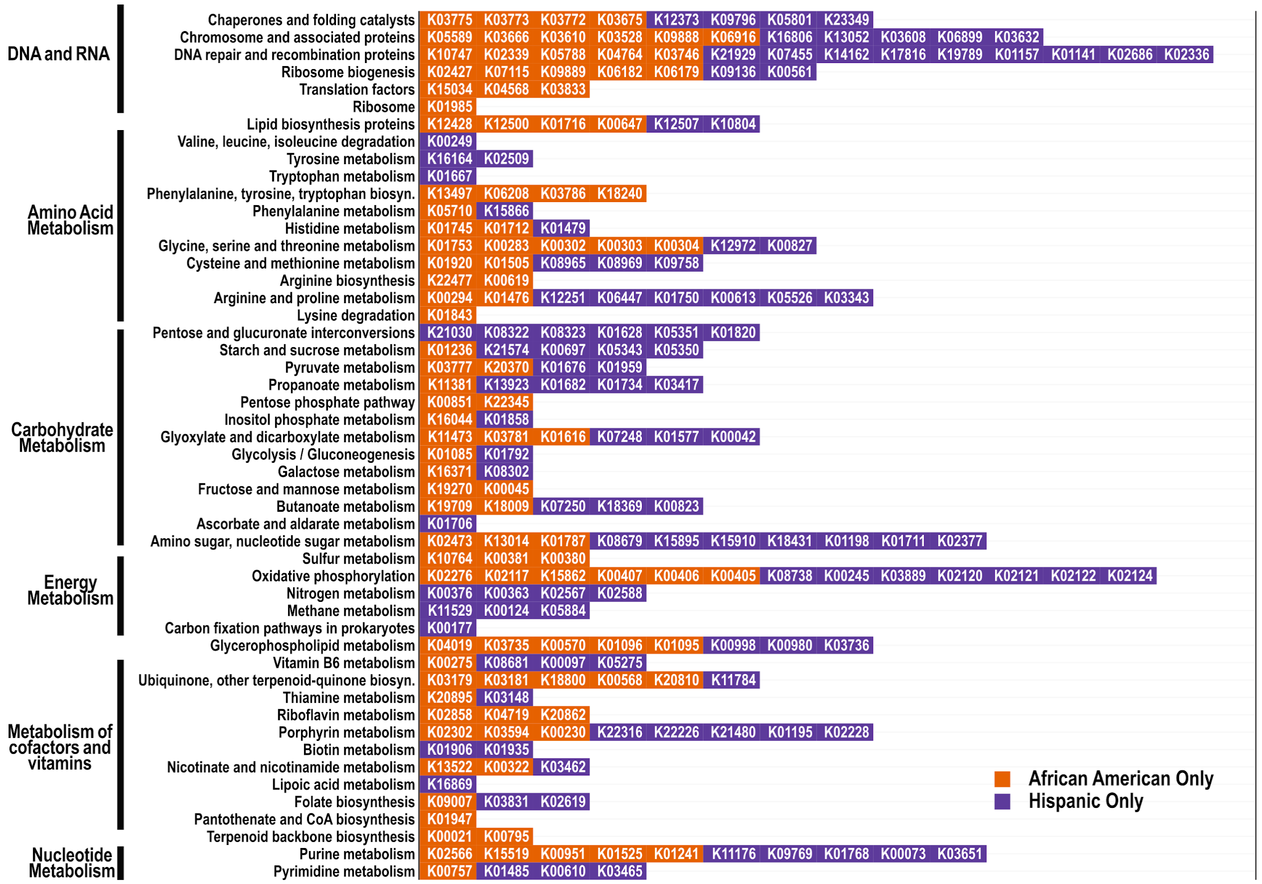

Figure S2. The African American and Latin American Hispanic caries samples showed differences in the functional pathways that were differentially expressed. The figure shows KEGG orthologs (KO numbers) that were significantly differentially expressed in only one group or the other.

Table S1: Subject Metadata

Subject ID	Cohort	Sex	Race/Ethnicity	Age	Dentition	Caries	dmft	dmfs
63	ECC	F	African American	5	Primary	Y	9	16
65	ECC	F	African American	4	Primary	Y	16	33
68	ECC	M	African American	3	Primary	Y	2	6
75	ECC	F	African American	5	Early mixed	Y	5	11
76	ECC	M	African American	5	Primary	Y	11	34
77	ECC	M	African American	5	Primary	Y	11	38
81	ECC	F	African American	3	Primary	Y	7	12
84	ECC	F	African American	5	Early mixed	Y	5	9
87	ECC	M	African American	4	Primary	Y	4	8
89	ECC	F	African American	4	Primary	Y	5	9
90	ECC	F	African American	4	Primary	Y	2	2
93	ECC	F	African American	5	Early mixed	Y	4	6
67	ECC	F	Latin American Hispanic	4	Primary	Y	7	24
71	ECC	M	Latin American Hispanic	5	Primary	Y	16	44
72	ECC	F	Latin American Hispanic	5	Primary	Y	9	23
74	ECC	M	Latin American Hispanic	4	Primary	Y	11	22
80	ECC	M	Latin American Hispanic	3	Primary	Y	8	9
83	ECC	F	Latin American Hispanic	5	Primary	Y	15	30
95	ECC	M	Latin American Hispanic	5	Primary	Y	9	24

Table S2: Sequencing depth for caries and non-caries plaque samples from ECC cohort.

Sample Name	Subject ID	Designation	Cohort	Site	Study	Read Count	Reads after human subtraction	% non human
63RNC-2	63	RNC	ECC	Non-Disease	Caries_AA	87971650	75021880	85.27961
63RC-1	63	RC	ECC	Disease	Caries_AA	85896308	83559039	97.2789645
65RC-1	65	RC	ECC	Disease	Caries_AA	82616744	80903453	97.9262182
65RNC-1	65	RNC	ECC	Non-Disease	Caries_AA	82845122	81526021	98.4077506
67RC-1	67	RC	ECC	Disease	Caries_Hisp	94774352	93750873	98.9200886
67RNC-1	67	RNC	ECC	Non-Disease	Caries_Hisp	79607664	77419378	97.2511617
68RC-1	68	RC	ECC	Disease	Caries_AA	86957218	84195243	96.8237542
68RNC-1	68	RNC	ECC	Non-Disease	Caries_AA	113240442	110890657	97.9249595
71RC-1	71	RC	ECC	Disease	Caries_Hisp	107140454	104111770	97.1731649
71RNC-2	71	RNC	ECC	Non-Disease	Caries_Hisp	104365060	87444452	83.787095
72RC-1	72	RC	ECC	Disease	Caries_Hisp	99968812	94937303	94.9669213
72RNC-2	72	RNC	ECC	Non-Disease	Caries_Hisp	116337046	114777008	98.6590359
74RC-1	74	RC	ECC	Disease	Caries_Hisp	99068026	97224560	98.1391918
74RNC-1	74	RNC	ECC	Non-Disease	Caries_Hisp	112620506	111304994	98.8319072
75RC-1	75	RC	ECC	Disease	Caries_AA	93454296	75863200	81.1767926
75RNC-1	75	RNC	ECC	Non-Disease	Caries_AA	102733062	100087351	97.4246743
76RC-3	76	RC	ECC	Disease	Caries_AA	101576372	100464467	98.9053507
76RNC-3	76	RNC	ECC	Non-Disease	Caries_AA	81927282	76681071	93.5965031
77RC-1	77	RC	ECC	Disease	Caries_AA	88348882	87314417	98.8291137
77RNC-1	77	RNC	ECC	Non-Disease	Caries_AA	106216902	105085083	98.9344267
80RC-1	80	RC	ECC	Disease	Caries_Hisp	93243222	91838861	98.4938734
80RNC-1	80	RNC	ECC	Non-Disease	Caries_Hisp	99020386	97796493	98.763999
81RC-1	81	RC	ECC	Disease	Caries_AA	80165936	77661130	96.8754734
81RNC-1	81	RNC	ECC	Non-Disease	Caries_AA	85284476	81446959	95.5003335
83RC-2	83	RC	ECC	Disease	Caries_Hisp	62522952	60854310	97.3311529
83RNC-1	83	RNC	ECC	Non-Disease	Caries_Hisp	116512996	105577834	90.6146418
84RC-1	84	RC	ECC	Disease	Caries_AA	107731346	105135380	97.5903336
84RNC-1	84	RNC	ECC	Non-Disease	Caries_AA	91731270	90495281	98.652598
87RC-1	87	RC	ECC	Disease	Caries_AA	99836458	95567577	95.7241262
87RNC-1	87	RNC	ECC	Non-Disease	Caries_AA	104978292	103064408	98.1768764
89RC-1	89	RC	ECC	Disease	Caries_AA	115838160	105062051	90.697272
89RNC-1	89	RNC	ECC	Non-Disease	Caries_AA	130449774	126302611	96.8208738
90RC-2	90	RC	ECC	Disease	Caries_AA	127909718	125893951	98.4240705
90RNC-1	90	RNC	ECC	Non-Disease	Caries_AA	119501094	107024763	89.5596512
93RC-1	93	RC	ECC	Disease	Caries_AA	89313918	83667750	93.6782888
93RNC-1	93	RNC	ECC	Non-Disease	Caries_AA	94077082	92451842	98.2724379
95RC-1	95	RC	ECC	Disease	Caries_Hisp	95671398	91220312	95.347527
95RNC-2	95	RNC	ECC	Non-Disease	Caries_Hisp	130322150	128710777	98.7635463

Table S3: Consistently dominant taxa in ECC cavity microbiome vs paired non-cavity plaque samples.

Rank by	Species Name	Up in X	Median Fold	Median
1	*Streptococcus mutans*	13	495.6212	9.05505
2	*Parascardovia denticolens*	11	156.2056	0.6716
3	*Olsenella sp. oral taxon 807*	10	194.9256	1.99417
4	*Olsenella uli*	9	Inf	0.22583
5	*Selenomonas sputigena*	9	80.47685	1.21995
6	*Prevotella denticola*	9	16.24448	1.73843
7	*Lactobacillus rhamnosus*	8	Inf	4.43949
8	*Atopobium parvulum*	8	260.6531	0.73508
9	*Selenomonas sp. oral taxon 136*	8	41.00642	1.39458
10	*Prevotella melaninogenica*	8	32.45457	0.51339
11	*Veillonella atypica*	8	18.32311	0.9471
12	*Lactobacillus salivarius*	7	Inf	0.28098
13	*Cryptobacterium curtum*	7	Inf	0.1823
14	*Streptococcus parasanguinis*	7	281.4632	2.30288
15	*Streptococcus salivarius*	7	140.914	0.95304
16	*Campylobacter gracilis*	7	80.58658	0.35606
17	*Propionibacterium australiense*	7	63.4081	0.27486
18	*Actinomyces sp. oral taxon 414*	7	13.39429	5.34545
